# Supplementary material for: Plasma Level of Circular RNA hsa_circ_0000190 Correlates with Tumor Progression and Poor Treatment Response in Advanced Lung Cancers
Source: Cancers (Basel). 2020 Jun 30;12(7):1740. doi: 10.3390/cancers12071740 (PMC7408140; doi:10.3390/cancers12071740)
Supplement: Supplementary file 1 [file cancers-12-01740-s001.zip › cancers-830480 supplementary revised.docx]

**Supplementary Figures and Tables**

**
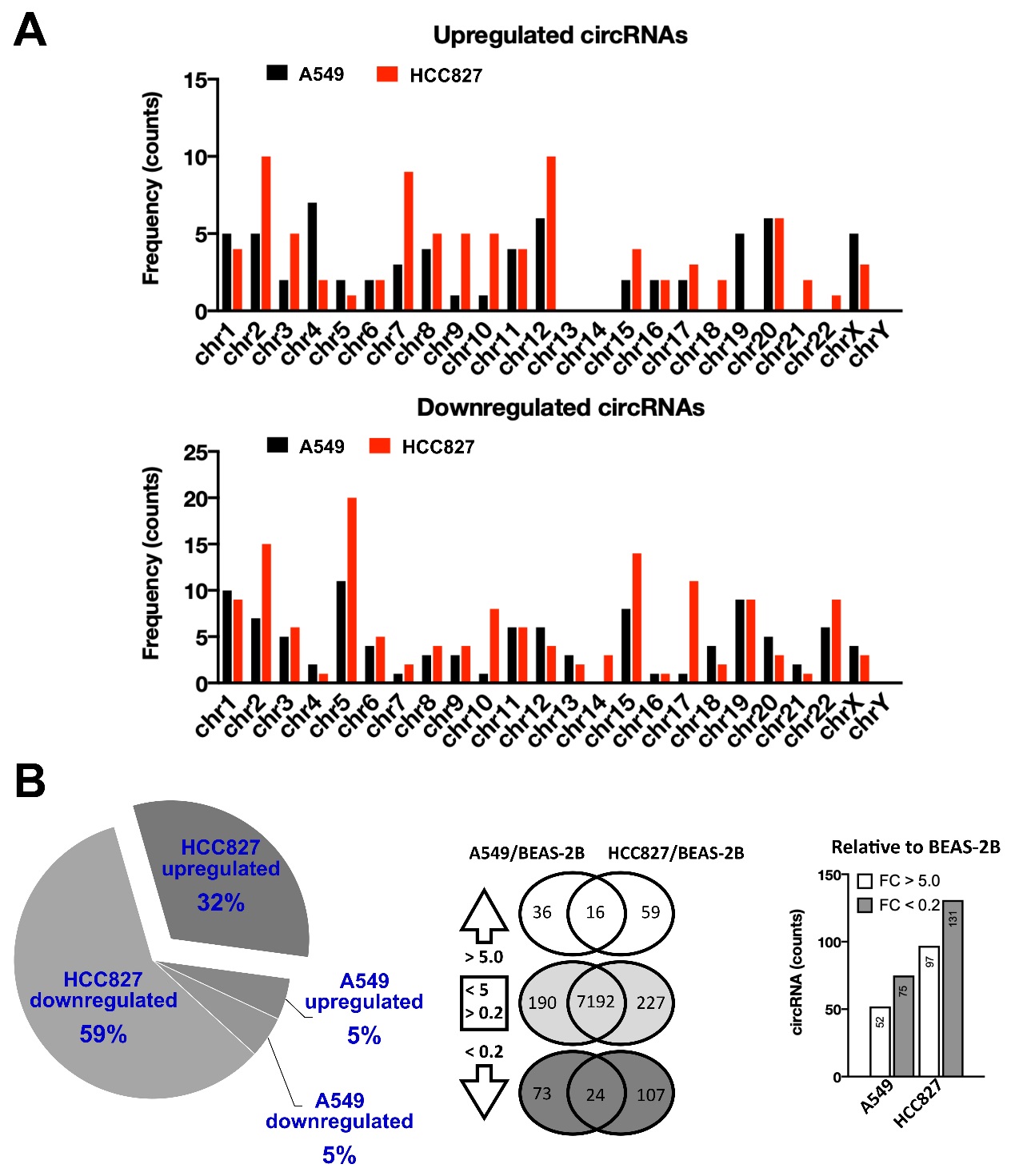
**

**Supplementary Figure 1. RNA-Seq analysis­ of circRNA transcriptomes of LC cell lines.** (**A**) Distribution of circRNAs identified to be expressed in A549 and HCC827 cell lines across the chromosomes of the human genome. (**B**) Essential statistics showing the percentages (left panel) and the numbers of common and unique (middle and right panels) circRNAs differentially expressed in A549 and HCC827 cell lines as compared to BEAS-2B with the indicated fold change (FC) cut-offs.


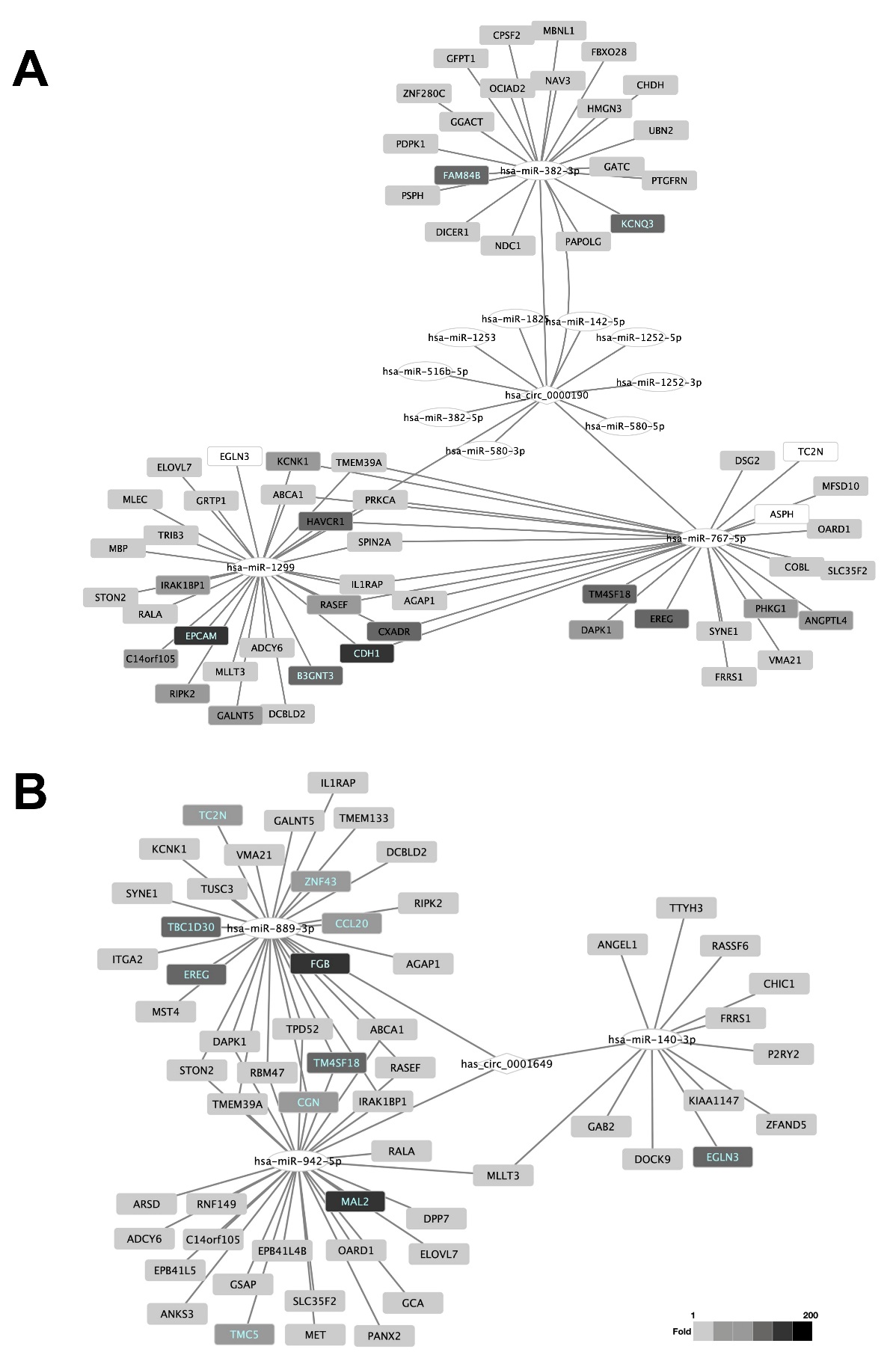


**Supplementary Figure 2. Predicted downstream network of hsa_circ_0000190 and hsa_circ_0001649.** (**A,B**) The downstream network regulated by hsa_circ_0000190 (**A**) and hsa_circ_0001649 predicted by the integrative analysis of the CircInteractome and TargetScan databases.

|  | **Table S1: Patient characteristics of stage IIIA-IV lung cancer patients** | | | |
| --- | --- | --- | --- | --- |
|  |  | | | Total no. |
|  | Patient no. | | | 166 |
|  | Gender (%) | | |  |
|  | Male | | | 122 (73.5) |
|  | Female | | | 44 (26.5) |
|  | Mean age (range, yr) | | | 63.5 (35-90) |
|  | With smoking history | | | 90 |
|  | Performance status (ECOG) (%) | | |  |
|  | 0 | | | 79 (47.6) |
|  | 1 | | | 69 (41.6) |
|  | 2 | | | 18 (10.8) |
|  | Lung cancer staging (%) | | |  |
|  | IIIa | | | 11 (6.6) |
|  | IIIb | | | 11 (6.6) |
|  | IV | | | 144 (86.8) |
|  | Histology (%) | | |  |
|  | Adenocarcinoma | | | 130 (78.3) |
|  | Squamous cell carcinoma | | | 19 (11.5) |
|  | Non-adeno/non-sqcc NSCLC | | | 15 (9) |
|  | SCLC | | | 2 (1.2) |
|  | Patients who received immunotherapy (n=50) | | |  |
|  | 1^st^ line treatment | | | 11 (22) |
|  | 2^nd^ line treatment | | | 13 (26) |
|  | 3^rd^ line treatment | | | 18 (36) |
|  | 4^th^ line treatment | | | 4 (8) |
|  | 5^th^ line treatment | | | 1 (2) |
|  | 6^th^ line treatment | | | 1 (2) |
|  | 7^th^ line treatment | | | 2 (4) |
|  | Patients who received chemotherapy (n=133) | | |  |
|  | Pemetrexed | | | 83 (62.4) |
|  | Docetaxel | | | 28 (21.1) |
|  | Paclitaxel | | | 32 (24.1) |
|  | Gemcitabine | | | 19 (14.3) |
|  | Patients who received targeted therapy (n=93) | | |  |
| Gefitinib | | | | 28 (30.1) |
| Erlotinib | | | | 36 (38.7) |
| Afatinib | |  |  | 31 (33.3) |
